# Comparison of presepsin and Mid-regional pro-adrenomedullin in the diagnosis of sepsis or septic shock: a systematic review and meta-analysis

**DOI:** 10.1186/s12879-023-08262-4

**Published:** 2023-05-05

**Authors:** Jun Liang, Yingli Cai, Yiming Shao

**Affiliations:** 1 Department of Emergency, the First People’s Hospital of Zhaoqing, Zhaoqing City, China; 2 Jinan University, No.601, West Huangpu Avenue, Guangzhou, 510632 China

**Keywords:** MR-proADM, Presepsin, Sepsis, Septic shock, Diagnosis, Meta-analysis

## Abstract

**Background:**

The early diagnosis of sepsis is hampered by the lack of reliable laboratory measures. There is growing evidence that presepsin and Mid-regional pro-adrenomedullin (MR-proADM) are promising biomarkers in the diagnosis of sepsis. This study was conducted to evaluate and compare the diagnostic value of MR-proADM and presepsin in sepsis patients.

**Methods:**

We searched Web of Science, PubMed, Embase, China national knowledge infrastructure, and Wanfang up to 22th July, 2022, for studies evaluating the diagnosis performance of presepsin and MR-proADM in adult sepsis patients. Risk of bias was assessed using quadas-2. Pooled sensitivity and specificity were calculated using bivariate meta-analysis. Meta-regression and subgroup analysis were used to find source of heterogeneity.

**Results:**

A total of 40 studies were eventually selected for inclusion in this meta-analysis, including 33 for presepsin and seven for MR-proADM. Presepsin had a sensitivity of 0.86 (0.82–0.90), a specificity of 0.79 (0.71–0.85), and an AUC of 0.90 (0.87–0.92). The sensitivity of MR-proADM was 0.84 (0.78–0.88), specificity was 0.86 (0.79–0.91), and AUC was 0.91 (0.88–0.93). The profile of control group, population, and standard reference may be potential sources of heterogeneity.

**Conclusions:**

This meta-analysis demonstrated that presepsin and MR-proADM exhibited high accuracy (AUC ≥ 0.90) in the diagnosis of sepsis in adults, with MR-proADM showing significantly higher accuracy than presepsin.

**Supplementary Information:**

The online version contains supplementary material available at 10.1186/s12879-023-08262-4.

## Background

Sepsis, a complex disorder which progresses as a dysregulated host response to infections [1], is a major challenge in emergency departments and intensive care units. Despite progress in clinical support has been made through advances in antibacterial therapy, sepsis and its sequelae are still associated with a high risk of death [2, 3]. The accurate and rapid diagnosis of sepsis is often difficult in clinical practice as its clinical manifestation may be confused with other normal inflammatory response of uncomplicated infection [4], as well as the lack of diagnostic tools. A delayed diagnosis may result in a more serious condition, such as multiple system organ failure. Therefore, it is necessary to develop a reliable method to improve the diagnosis of sepsis.

Clinical decisions for sepsis treatment are usually based on the physician's experience due to the lack of rapid and accurate diagnosis tools. Although blood culture is commonly regarded as the "gold standard" for diagnosis of sepsis, it takes several days to obtain results and often produces false negative (FN) results due to the use of antibiotics. Moreover, false positive (FP) results may occur due to sample contamination [5]. The use of biomarkers can greatly improve a physician's ability to accurately diagnose sepsis and initiate appropriate treatment. C-reactive protein (CRP) and procalcitonin (PCT) are among the most extensively studied biomarkers for sepsis diagnosis. However, the accuracy of sepsis prediction by CRP is limited by its low sensitivity [6], and the variation in reported cutoff values among studies greatly hinders the practical application of PCT in clinical settings [7]. An ideal biomarker with adequate clinical accuracy for the diagnosis of sepsis is still needed.

Presepsin, also named as soluble CD14 subtype, is a N-terminal fragment of soluble CD14, which is released from the surface of immune cell lines after stimulation by pathogens. Serum presepsin can be easily detected [8]. Level of presepsin increases within 2 h after the onset of infection and peaks at 3 h [8]. The quick detection makes it a potential candidate biomarker for sepsis. However, the interpretation of elevated presepsin level requires special caution in several clinical conditions. Age (newborns and the elderly), acute pancreatitis, and burns can influence presepsin levels [9–11]. Furthermore, since presepsin is filtered by the glomerulus and reabsorbed by the proximal tubules, any condition that affects kidney filtrating function will have an impact on plasmatic presepsin. Mid-regional pro-adrenomedullin (MR-proADM), a peptide fragment of hormone adrenomedullin (ADM), has recently emerged as a promising diagnostic biomarker in the evaluation of sepsis. ADM is widely expressed in many organs and tissues. In healthy subjects, the plasma concentration of ADM is low, while during pathological events, the concentration is significantly increased. Changes in plasma concentration are proportional to the severity of the disease [12]. Since ADM is rapidly cleared from the circulation which makes it hard to be detected, more stable MR-proADM directly reflects the level of ADM and is therefore used as an alternative. Burns are also associated with increased levels of MR-proADM [13].

The performance of the two novel biomarkers were unclear. Therefore, the objective of this meta-analysis was to determine and compare the diagnostic performance of presepsin and MR-proADM in sepsis.

## Methods

This study followed the Preferred Reporting Items for a Systematic Review and Meta-analysis of Diagnostic Test Accuracy Studies (PRISMA-DTA) [14]. The protocol was registered on PROSPERO with reference number CRD42022357335.

### Search strategy

We searched Web of Science, PubMed, Embase, China national knowledge infrastructure (CNKI) and Wanfang up to 22th July, 2022. S1 table shows the complete search strategy. Briefly, population (adults with sepsis or septic shock), index test (MR-proADM and presepsin), comparison (adults not suffering from sepsis or septic shock), and outcome (diagnostic accuracy) were used.

### Study selection

A study was selected if it satisfied the following criteria: (1) purpose of the study was to evaluate diagnosis performance of presepsin or MR-proADM in sepsis or septic shock. (2) adult patients with sepsis or septic shock were included in the experimental group and patients with non-sepsis or healthy participants were in the control group; (3) a gold standard was clearly defined for the diagnosis of sepsis. The exclusion criteria were as follows: (1) there is no enough data to calculate diagnostic accuracy estimates; (2) conference abstracts, reviews, and editorials. Two independent reviewers (JL and YC) completed the study screening with disagreement resolved by consensus.

### Data extraction

We extracted the following data by two independent reviewers (JL and YC): first author, year of publication, study design, region, sample size, severity of patient, sample type, assay methodology, standard reference, cut-off value, area under the curve (AUC), sensitivity, specificity. Disagreement was resolved by consensus.

### Quality assessment

Quality Assessment Tool for Diagnostic Accuracy Studies (QUADAS-2 score) was used for quality assessment [15]. Risk of bias domains, including patient selection, index test, reference standard, flow and timing, and applicability concerns were evaluated. Two independent reviewers (YC and YS) completed the quality assessment with disagreement resolved by consensus.

### Statistical analysis

The heterogeneity caused by non-threshold effects was measured using bivariate boxplot and *I*^2^. If *I*2 ≥ 50%, *P* values ≤ 0.05, or studies fall outside the bivariate boxplot, indicating significant heterogeneity due to non-threshold effects, then meta-regression or subgroup analysis is performed to identify the source of heterogeneity. Sensitivity, specificity, positive likelihood ratio (PLR), negative likelihood ratio (NLR), diagnostic ratio (DOR), AUC, and corresponding 95% confidence interval (CI) were calculated using a binary regression model of STATA 15.1 software using true positive (TP), FP, FN, and true negative (TN). Literature quality evaluation using Revman 5.4.1. Deek funnel diagrams were used to detect publication bias, with *P* < 0.05 indicating publication bias in the study.

## Results

### Characteristics of the included studies

**Fig. 1:**
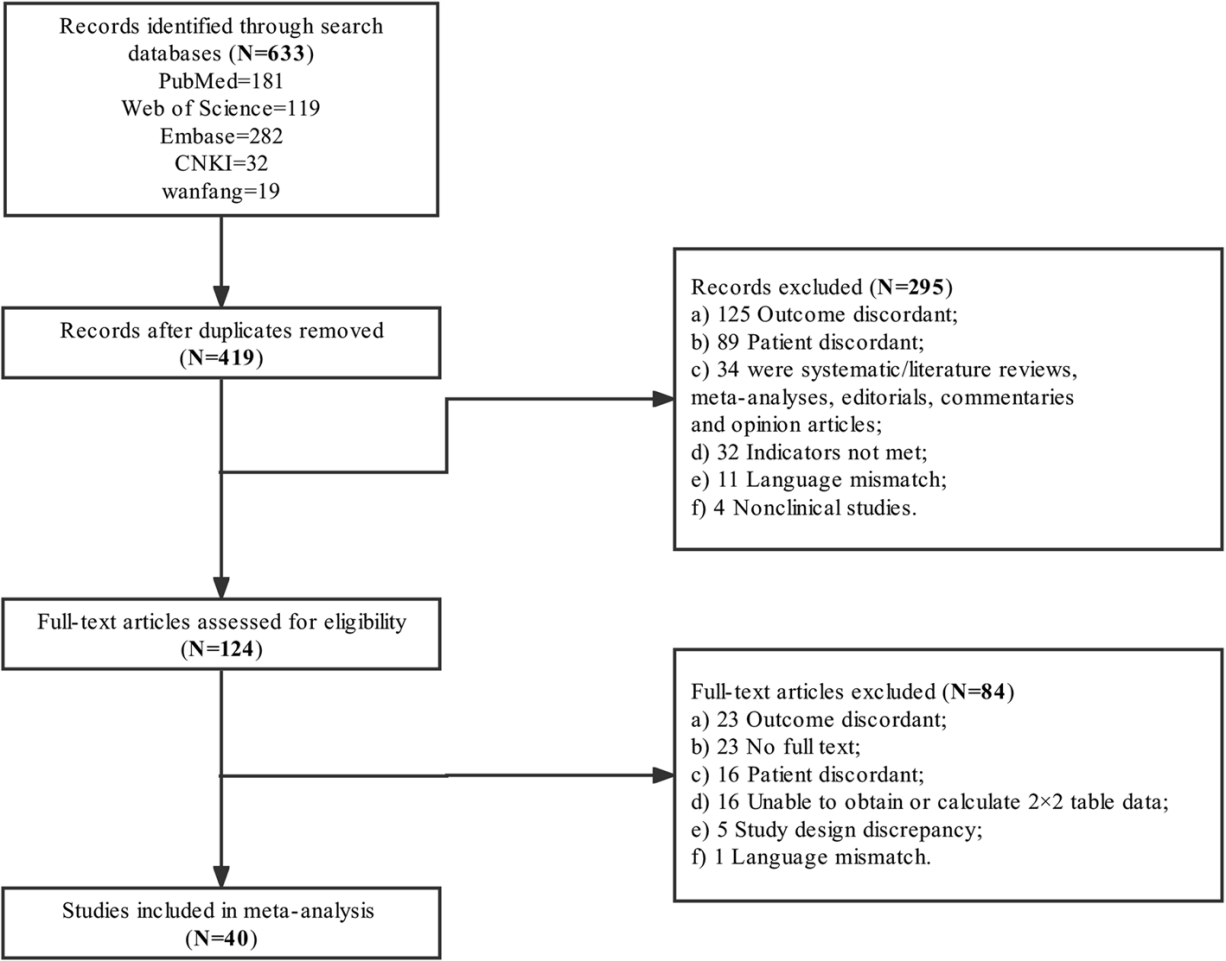
Flow diagram for study selection

A total of 419 studies were included after the database search. Of these, 295 studies were excluded by the abstract screening, and 85 were excluded by the full-text screening (Fig. 1). Finally, 40 studies were included in this meta-analysis, with 33 for the diagnosis of presepsin and seven for MR-proADM (Table 1) [16–54].

**Table 1:** Characteristics of the included studies

Author, year	Biomarker	Study design	Region	Control group	Sample	Standard reference	Assay method	Cut off value (pg/mL)
Lee, 2022 [16]	Presepsin	Prospective	Korea	Non-sepsis	Plasma	Sepsis-3	CLEIA	582
Kang, 2022 [17]	Presepsin	Retrospective	Korea	Non-sepsis	Unknown	Sepsis-2	Unknown	671.5
Jeong, 2022 [18]	Presepsin	Prospective	Korea	Non-sepsis	Plasma	Sepsis-3	CLEIA	314
Tahmaz, 2022 [19]	Presepsin	Prospective	Turkey	Healthy	Plasma	Sepsis-3	CLEIA	439
Chen, 2021 [20]	Presepsin	Prospective	China	Non-sepsis	Plasma	Sepsis-3	CLEIA	404.5
Yonaha, 2021 [21]	Presepsin	Prospective	Japan	Non-sepsis	Plasma	Sepsis-3	CLEIA	1315
Abdelshafey, 2021 [22]	Presepsin	Prospective	Unknown	Non-sepsis	Complete blood	Sepsis-3	CLEIA	640
Liu, 2020 [23]	Presepsin	Prospective	China	Non-sepsis	Serum	Sepsis-3	ELISA	89.26
Yamamoto, 2019 [24]	Presepsin	Prospective	Japan	Non-sepsis	Unknown	Sepsis-3	Unknown	557
Nakamura, 2019 [25]	Presepsin	Retrospective	Japan	Non-sepsis	Unknown	Sepsis-3	CLEIA	240
JUROŠ, 2019 [26]	Presepsin	Prospective	Croatia	Non-sepsis	Unknown	Sepsis-3	CLEIA	349
Jereb, 2019 [27]	Presepsin	Prospective	Slovenia	Non-sepsis	Plasma	Sepsis-3	CLEIA	751.5
Lu, 2018 [28]	Presepsin	Prospective	China	Non-sepsis	Complete blood	ACCP/SCCM	CLEIA	407
Li, 2018 [29]	Presepsin	Unknown	China	Healthy	Complete blood	Sepsis-2	CLEIA	586.6
El-Shafie, 2017 [30]	Presepsin	Prospective	Egypt	Non-sepsis	Complete blood	Sepsis-2	CLEIA	422
Romualdo, 2017 [31]	Presepsin	Prospective	Spain	Non-sepsis	Plasma	Sepsis-3	CLEIA	312
Klouche, 2016 [32]	Presepsin	Prospective	France	Non-sepsis	Complete blood	Sepsis-1	CLEIA	466
Ali, 2016 [33]	Presepsin	Prospective	Egypt	Non-sepsis	Plasma	Sepsis-3	CLEIA	907
Amer, 2016 [34]	Presepsin	Prospective	Egypt	Healthy	Complete blood	SSCG 2012	CLEIA	455.5
Enguix-Armada, 2016 [35]	Presepsin	Prospective	Spain	Healthy	Plasma	SSCG 2012	CLEIA	101.6
Sato, 2015 [36]	Presepsin	Unknown	Japan	Healthy	Complete blood	SSCG 2012	CLEIA	569.5
Carpio, 2015 [37]	Presepsin	Prospective	Peru	Healthy	Plasma	Sepsis-1	CLEIA	370
Sargentini, 2015 [38]	Presepsin	Prospective	Italy	Healthy	Plasma	SSCG 2008	CLEIA	600
Popa, 2015 [39]	Presepsin	Retrospective	Thailand	Non-sepsis	Complete blood	Sepsis-3	CLEIA	380
Behnes, 2015 [40]	Presepsin	Prospective	Germany	Non-sepsis	Complete blood	Sepsis-1	CLEIA	530
Madenci, 2014 [41]	Presepsin	Prospective	Turkey	Non-sepsis	Plasma	Sepsis-2	CLEIA	542
Kweon, 2014 [42]	Presepsin	Prospective	Korea	Healthy	Complete blood	Sepsis-1	CLEIA	430
Liu, 2013 [43]	Presepsin	Prospective	China	Healthy	Complete blood	Sepsis-2	CLEIA	317
Vodnik, 2013 [44]	Presepsin	Prospective	Serbia	Healthy	Complete blood	Sepsis-1	CLEIA	630
Ulla, 2013 [45]	Presepsin	Prospective	Italy	Non-sepsis	Plasma	Sepsis-1	CLEIA	600
Shozushima, 2011 [46]	Presepsin	Prospective	Japan	Healthy	Plasma	Sepsis-1	CLEIA	399
Venugopalan, 2019 [47]	Presepsin	Prospective	India	Non-sepsis	Complete blood	Sepsis-2	ELISA	93.71
Li, 2016 [48]	Presepsin	Prospective	China	Non-sepsis	Serum	Sepsis-3	CLEIA	672.5
Martin-Fernandez, 2020 [49]	MR-proADM	Prospective	Spain	Healthy	Plasma	Sepsis-3	sandwich immunoassay	1.165
Enguix-Armada, 2016 [35]	MR-proADM	Prospective	Spain	Healthy	Plasma	SSCG 2012	sandwich immunoassay	1.11
Angeletti, 2015 [50]	MR-proADM	Unknown	Italy	Non-sepsis	Complete blood	Sepsis-1	immunoluminometric	1.06
Angeletti, 2013 [51]	MR-proADM	Prospective	Italy	Healthy	Plasma	Sepsis-2	immunoluminometric	0.8
Angeletti, 2015 [52]	MR-proADM	Retrospective	Italy	Non-sepsis	Plasma	SSCG 2012	immunoluminometric	1
Spoto, 2020 [53]	MR-proADM	Prospective	Italy	Healthy	Plasma	Sepsis-3	immunoluminometric	1.5
Spoto, 2018 [54]	MR-proADM	Prospective	Italy	Non-sepsis	Plasma	Sepsis-3	immunoluminometric	1.5

*ELISA* Enzyme linked immunosorbent assay, *CLEIA* Chemiluminescent enzyme immunoassay

In the 33 studies [16–48] evaluating the diagnostic performance of presepsin, 28 studies [16, 18–22, 24, 26–28, 30–35, 37, 38, 40–48] were prospective, three were retrospective, and two were unclear [29, 36]. Nineteen studies [16–21, 23–25, 28, 29, 36, 39, 41–43, 46–48] were conducted in Asia, nine in Europe [26, 27, 31, 32, 35, 38, 40, 44, 45], three in Africa [30, 33, 34], one in America [37], and one unclear [22]. Fifteen studies used Sepsis-3 as the standard reference for sepsis diagnosis [27, 29, 44–47].

In the seven studies [35, 49–54] evaluating the diagnostic performance of MR-proADM, five studies [35, 49, 51, 53, 54] were prospective, one [52] were respective, and one were unclear [50]. All the studies were conducted in Europe. Three studies [49, 53, 54] used Sepsis-3 as the standard reference for sepsis diagnosis.

### Results of quality assessment

We assessed the quality of the literature using QADAS 2 and the results are shown in Additional file 5. Red, yellow, and green indicate high, medium, and low risk classifications, respectively. The graph shows the risk assessment for each of the 40 studies. In terms of risk of bias, four studies [28, 31, 33, 44] had unspecified bias in patient selection; 21 studies [17–21, 23, 25, 27–30, 36, 40–42, 44, 48, 50–53] had unspecified bias in index testing; one study [17] was assigned high bias in terms of reference standard; one study [53] was judged to be highly biased in terms of flow and timing. In terms of suitability, two studies [24, 47] had high bias in patient selection; three studies [24, 47, 52] were judged to have high bias relative to the index test; one study [33] had a high risk of suitability evaluation for the gold standard. Overall quality was good.

### Diagnostic value of presepsin and MR-proADM

**Fig. 2:**
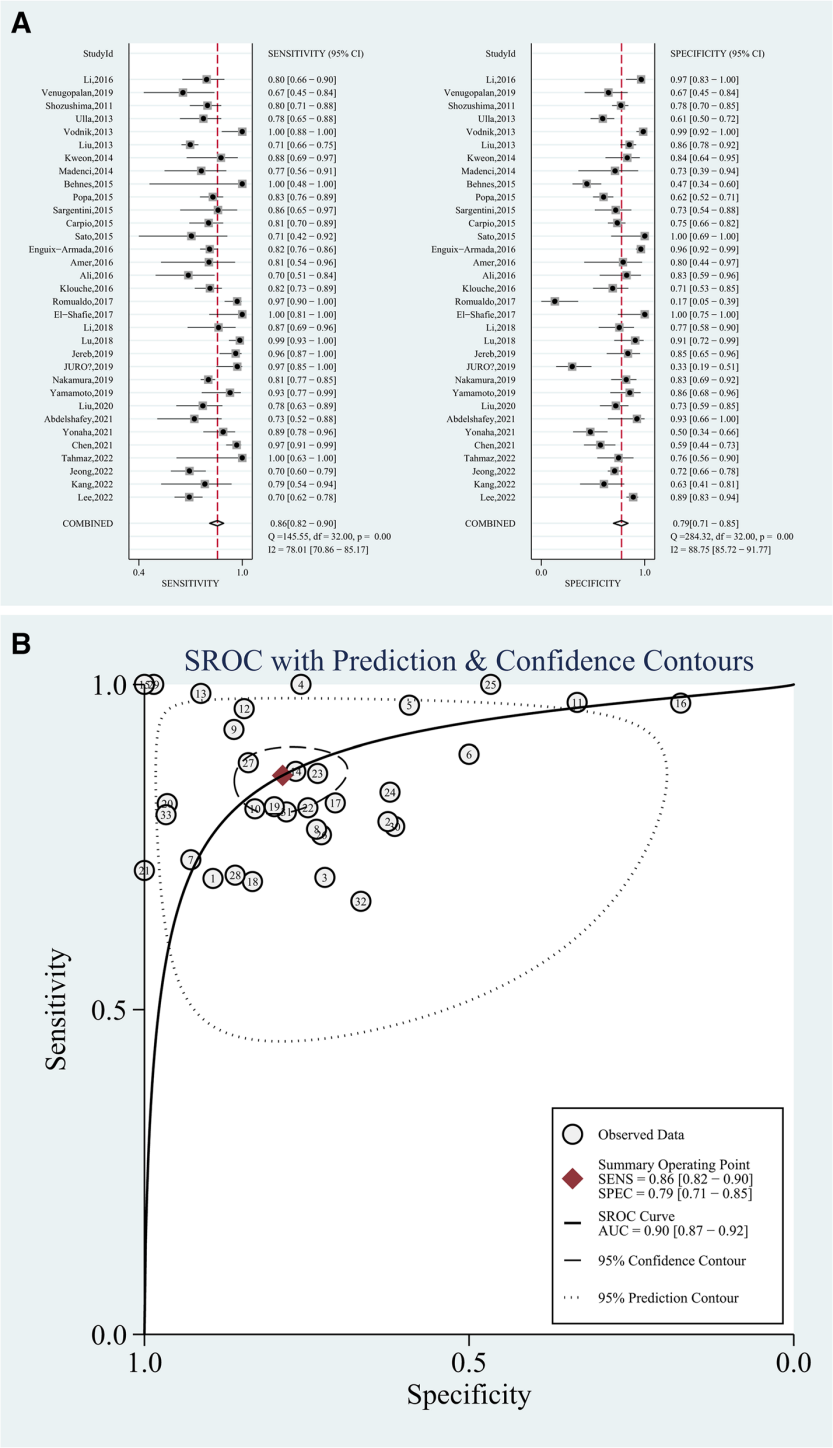
Forest plots (**A**) and ROC curve (**B**) for presepsin in the diagnosis of sepsis

**Fig. 3:**
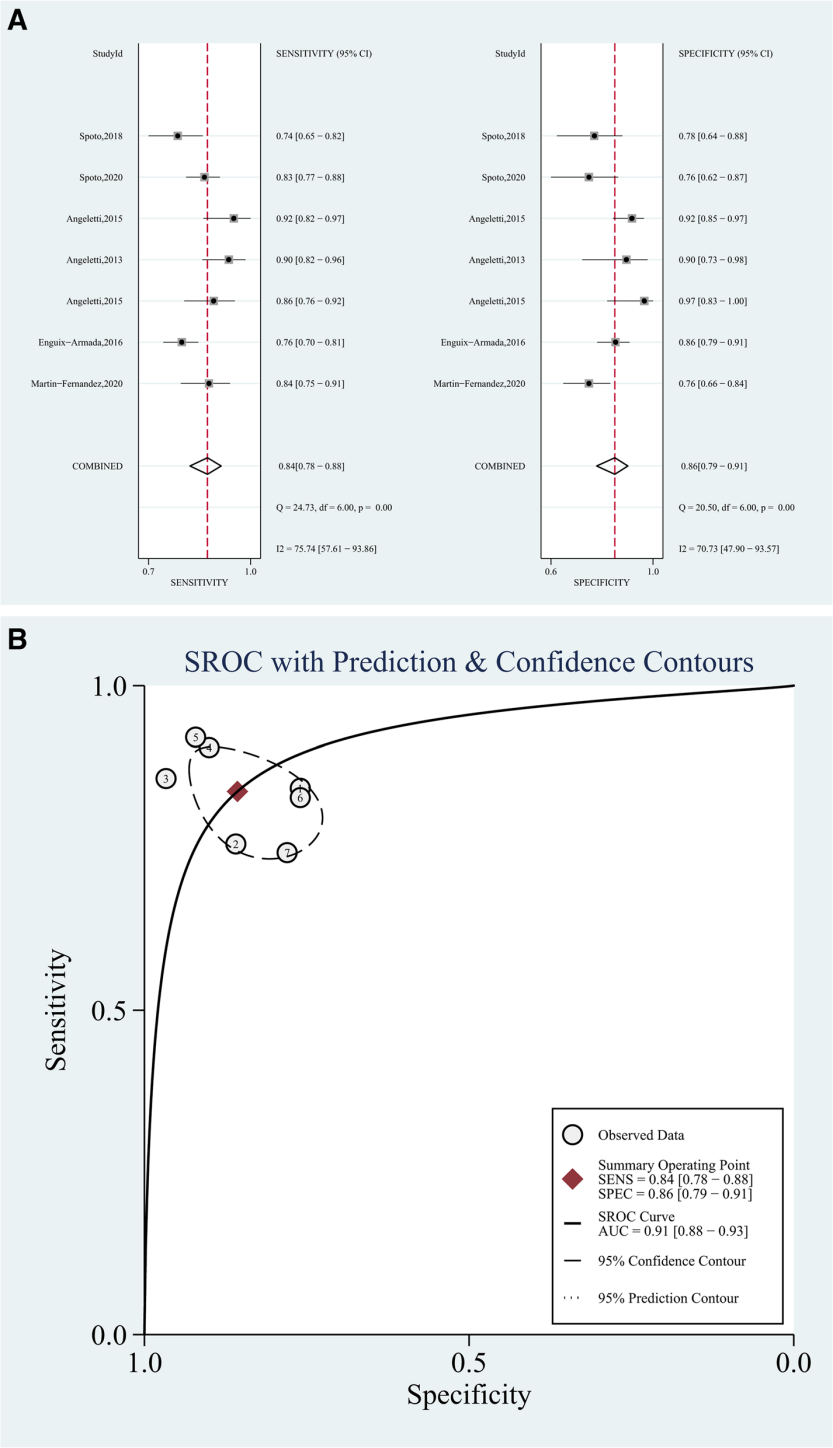
Forest plots (**A**) and ROC curve (**B**) for MR-pro-ADM in the diagnosis of sepsis

The forest plots and summary receiver operator characteristic curves (SROC) showing the diagnostic performance of biomarkers are illustrated in Fig. 2 for presepsin and Fig. 3 for MR-proADM. In the diagnosis of sepsis, presepsin achieved a pooled AUC of 0.90 (95% CI, 0.87–0.92), sensitivity of 0.86 (95% CI, 0.82–0.90), and specificity of 0.79 (95% CI, 0.71–0.85). For MR-proADM, the pooled AUC was 0.91 (95% CI, 0.88–0.93), sensitivity was 0.84 (95% CI, 0.78–0.88), and specificity was 0.86 (95% CI, 0.79–0.91).

Meanwhile, we compared presepsin with MR-proADM, and the results showed that the performance of the two biomarkers were statistically significant in terms of sensitivity, specificity and AUC (Table 2). The sensitivity of Presepsin was higher than MR-proADM (0.86 vs 0.84, *P* < 0.001), whereas the specificity of MR-proADM was higher than Presepsin (0.79 vs 0.86, *P* < 0.001). The AUC of MR-proADM was significantly higher than that of presepsin.

**Table 2:** Comparison of the diagnostic performance of presepsin with MR-proADM

Category	Sensitivity [95%CI]	*P*	Specificity [95%CI]	*P*	AUC [95%CI]	*P*	PLR [95%CI]	NLR [95%CI]	DOR [95%CI]
Presepsin	0.86 [0.82, 0.90]	/	0.79 [0.71, 0.85]	/	0.90 [0.87—0.92]	/	4.0 [3.0, 5.5]	0.18 [0.13, 0.23]	23 [14,36]
MR-proADM	0.84 [0.78, 0.88]	/	0.86 [0.79, 0.91]	/	0.91 [0.88—0.93]	/	5.8 [3.8, 9.0]	0.19 [0.14, 0.27]	31 [15,62]
Presepsin vs MR-proADM	0.86 vs 0.84	< 0.001	0.79 vs 0.86	< 0.001	0.90 vs 0.91	0.026	/	/	/

*PLR* Positive likelihood ratio, *NLR* Negative likelihood ratio, *DOR* Diagnostic odds ratio, *AUC* Area under curve

### Subgroup analysis of presepsin

For Presepsin, we performed subgroup analyses (Table 3) and meta-regression analyses (Figure S1). It was found that population characteristics of control group, region of the dataset, reference standard of sepsis, and cutoff value influenced the pooled sensitivity of the included studies and were the possible sources of heterogeneity. The sensitivity of presepsin was significantly higher in non-Asian populations than in Asian populations (0.90 vs. 0.84), and was significantly higher in studies using patients with non-sepsis disease as control group than healthy subjects as control group. Furthermore, studies using cutoff value < 445 ng/L showed slightly higher sensitivity than the studies with cutoff value greater than 445, while the difference in specificity between the two groups was not statistically significant.

**Table 3:** The result of meta-regression and Subgroup analysis for Presepsin

Category	NO.of studies	Sensitivity (95%CI)	*P*	*I*^*2*^	Specificity (95%CI)	*P*	*I*^*2*^
**Study design**							
Prospective	28	0.87 [0.83—0.91]	0.34	-	0.79 [0.72—0.86]	0.99	-
Retrospective	3	0.82 [0.67—0.96]	-	-	0.71 [0.45—0.96]	-	-
**Control group**							
Healthy volunteer	11	**0.85 [0.78—0.92]**	**0.00**	-	0.87 [0.79—0.94]	0.49	-
Non-sepsis	22	**0.87 [0.82—0.91]**	-	-	0.73 [0.65—0.82]	**-**	-
**Region**							
Asian	19	**0.84 [0.78—0.89]**	**0.00**	-	0.79 [0.71—0.88]	0.13	-
Other	13	**0.90 [0.85—0.95]**	-	-	0.76 [0.65—0.88]	**-**	-
**Cut-off value**							
> 455	17	0.85 [0.79—0.90]	**0.001**		0.82 [0.73—0.90]	**0.18**	
≤ 455	16	0.87 [0.82—0.92]			0.75 [0.65—0.86]		
**Assay**							
CLEIA	29	0.87 [0.83—0.91]	**0.80**	-	0.79 [0.72—0.86]	**0.79**	-
ELISA	2	0.73 [0.50—0.96]	-	-	0.71 [0.39—1.00]	-	-
**Standard Reference**							
Sepsis-3	15	0.84 [0.79—0.89]	**0.00**	**75.2%**	0.72 [0.62—0.80]	**0.00**	**85.2%**
Sepsis-2	6	0.76 [0.67—0.82]	0.165	36.3%	0.76 [0.65—0.85]	0.056	53.6%
Sepsis-1	7	0.82 [0.78—0.85]	0.551	0.0%	0.74 [0.62—0.83]	**0.00**	**84.2%**
SSCG 2012	3	0.81 [0.76—0.85]	0.542	0.0%	0.93 [0.78—0.98]	0.107	55.2%
SSCG 2008	1	0.86 [0.65—0.96]	-	-	0.73 [0.55—0.86]	-	-
**Sample**							
Plasma	2	0.83 [0.78—0.88]	**0.00**	**72.9%**	0.74 [0.65—0.81]	**0.00**	**85.6%**
Complete blood	3	0.82 [0.75—0.87]	**0.001**	**65.5%**	0.80 [0.70—0.88]	**0.00**	**79.1%**
Serum	1	0.79 [0.70—0.86]	0.791	0.0%	0.88 [0.43—0.99]	**0.028**	**79.3%**

*CLEIA* Chemiluminescent enzyme immunoassay, *ELISA* Enzyme linked immunosorbent assay

The sensitivity analysis in Figure S2 was performed to investigate the robust of our study. When seven outlier studies were excluded, the overall results were only minimally changed, suggesting that our results were not driven by these outlying points, which indicated that our study is robust. The bivariate box plot with most studies clustering within the median distribution suggested an acceptable degree of heterogeneity (Figure S3).

### Risk of publication bias

**Fig. 4:**
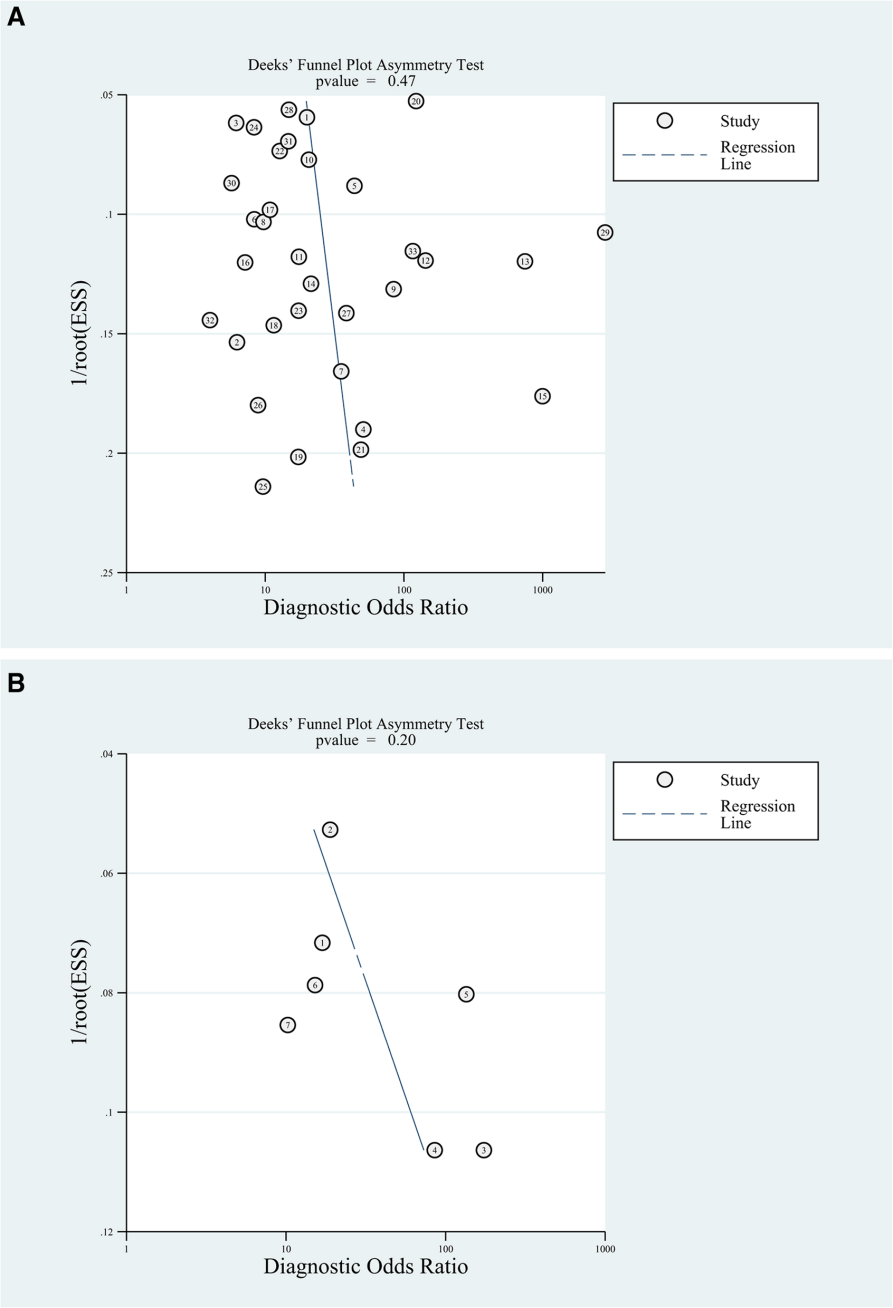
Publication bias for presepsin (**A**) and MR-pro-ADM (**B**)

We performed published bias analysis and the results (Fig. 4) showed no published bias.

## Discussion

This meta-analysis compared the performance of two novel biomarkers, presepsin and MR-proADM, in the diagnosis of sepsis. Both biomarkers showed good diagnostic performance, with AUC being 0.90 for presepssin and 0.91 for MR-proADM, proving their potential value in the assistance of sepsis diagnosis.

Clinically, early and accurate diagnosis of sepsis may be challenging. Conventional inflammatory biomarkers, such as CRP and PCT, have been extensively studied and applied in patients with sepsis. In several clinical studies, PCT was found to be more accurate than CRP in differentiating sepsis and SIRS [55, 56]. In a previous meta-analysis [57], the sensitivity and specificity were 0.80 (95% CI, 0.63–0.90) and 0.61 (95% CI, 0.50–0.72), respectively, for the diagnosis of sepsis using CRP, and 0.80 (95% CI, 0.69–0.87) and 0.77 (95% CI, 0.60–0.88), respectively, using PCT. In this study, sensitivity is 0.86 for presepsin and 0.84 for MR-proADM, and specificity is 0.79 and 0.86, respectively. Compared to PCT, the two novel biomarkers showed higher diagnostic accuracy. In addition, presepsin levels rise earlier than CRP or PCT in response to sepsis [58], which indicated their potential values as biomarkers for early diagnosis of sepsis. In addition, it has been reported that MR-proADM levels are not affected by the type of pathogen involved compared with PCT, but instead reflect the degree of organ failure and disease severity [59]. In contrast, previous studies have demonstrated that levels of PCT in patients with sepsis vary depending on the type of pathogen, with the highest levels observed in cases of gram-negative infections and lower levels seen in cases of yeast and intracellular germs [60, 61]. MR-proADM could provide broader and more reliable diagnostic and prognostic information.

The cause of sepsis should be considered for better diagnosis and management. Presepsin and MR-proADM are biomarkers that have been shown to be elevated in patients with sepsis triggered by bacterial infections. However, bacteria are not the only causative microbial pathogens. The diagnostic accuracy for sepsis caused by viruses, parasites, and fungi has not been well established yet. Angeletti et al. [51] conducted a study on a cohort of 200 patients with sepsis, categorized based on the source of the infection (Gram-positive and Gram-negative bacteria, yeast, or multiple microorganisms). The results showed that the MR-proADM had high diagnostic accuracy, with an AUC of 0.96 (*p* < 0.01), 0.94 (*p* < 0.001), 0.99 (*p* < 0.001), and 0.98 (*p* < 0.001) for the respective group. This study proved the potential value of MR-proADM in the diagnosis of sepsis caused by fungus. Additionally, presepsin levels were found to be elevated in patients with fungemia [62] and viral meningitis [63]. MR-proADM levels were found to be elevated in diseases caused by viral and fungal infections, such as invasive fungal diseases [64], COVID-19 [65], dengue hemorrhagic fever [66], and influenza A pneumonia [67]. Therefore, we recommended more studies investigate the value of presepsin and MR-proADM on the diagnosis of sepsis caused by pathogens other than bacteria, such as viruses, fungus and parasites.

Cut off values of presepsin for diagnosing sepsis are not always consistent among studies despite using the same assay, which hinders greatly in the clinical application of these biomarkers. In our study, the cut-off values reported range from 89.26 to 1,315 pg/mL for presepsin and 0.8 to 1.165 pg/mL for MR-proADM. Reasons for the wide range of cut off values may be differences in sepsis severity, study design, clinical settings, and type of samples. Specifically, patient inclusion criteria may be one of the main reason accounting for this discrepancy. It has been reported that in cases of chronic renal failure, resuscitations and trauma, presepsin levels may be falsely elevated [68]. Therefore, the effects of comorbidities on presepsin levels should be considered in future study to confirm a clinically useful cutoff value.

We observed heterogeneity among the included studies for the following possible reasons: First, the standard definition of sepsis has been updated thrice over the past decades, which results in the different reference standards used across studies. The latest international consensus on sepsis, sepsis-3, was developed to further refine sepsis, with a greater focus on identifying organ dysfunction in infection-related situations [69]. The broad definition of sepsis is a common limitation in all studies regarding the diagnosis of sepsis. Second, non-sepsis patients and healthy volunteers were evaluated as control groups, which may contribute to higher pooled outcomes than the real results [70]. Furthermore, some studies identify sepsis only through blood culture, microscopy or positive PCR, while others comprehensively assess patients and combine clinical, radiological and laboratory data. Taken together, individual patient differences, criteria for sepsis diagnosis, methods for testing samples, laboratory levels of testing, and differences in the instruments used are possible sources of heterogeneity. Under this consequence, it is recommended that the more rigorous study design, including but not limited to the procedure of patient selection, and timing of measurement, be applied in future work investigating the diagnostic value of biomarkers for sepsis to improve their validity.

Considering the good performance observed in this study, presepsin and MR-proADM can serve as candidate biomarkers for sepsis. However, several aspects need to be further explored to fully verify their utility in clinical practice. First, prospective studies with large sample size is needed. Second, antibiotic treatment lowers the serum level of presepsin [71–73]. It is necessary to consider this factor and perform sequential measurements of serum presepsin during the period of treatment as limited medicine information of patients is recorded in previous studies. Additionally, previous studies have proved the potential valued of presepsin as a reliable biomarker for antibiotic stewardship. A study conducted by Masson et al. [74] indicated that monitoring presepsin levels during the initial week of treatment could serve as a reliable indicator of the effectiveness of the antimicrobial treatment. A recent multicenter, prospective cohort trial revealed that the use of presepsin to guide antibiotic prescriptions in sepsis patients resulted in significant reductions in the duration of antibiotic treatment, ICU or hospital stay, and hospitalization costs, without increasing mortality rates, recurrent infections, or the risk of worsening organ failure [75]. Therefore, presepsin may not only be a promising biomarker for the diagnosis of sepsis, but also the management of antibiotic therapy. Finally, combination of the two biomarkers may be considered in future researches to achieve better diagnosis performance.

To the best of our knowledge, this is the first meta-analysis comparing the diagnostic performance of presepsin and MR-proADM in sepsis. The effects of several possible confounders on the outcome were evaluated by subgroup analysis. Nevertheless, several limitations exist. Firstly, only 7 studies evaluating the diagnostic value of MR-proADM were included in this study, as there was relatively little data available. Secondly, inclusion and exclusion criteria vary largely among the included studies. The above factors may cause risk of bias to the results of the present study.

## Conclusion

This meta-analysis indicated that both presepsin and MR-proADM exhibited high accuracy for the diagnosis of sepsis in adults. MR-proADM is significantly more accurate than presepsin. Future clinical trials are necessary to further verify their utility in clinical practice.

## Supplementary Information

**Additional file 1: Figure S1.** The result of meta-regression for presepsin.

**Additional file 2: Figure S2.** Graphical depiction of residual-based goodness-of-fit, bivariate normality, influence and outlier detection analyses.

**Additional file 3: Figure S3.** Bivariate boxplot for presepsin (A) and MR (B).

**Additional file 4: Table S1.** Search strategy used in this study

**Additional file 5:** Quality assessment of the included studies.

## Data Availability

The datasets used and/or analysed during the current study are available from the corresponding author on reasonable request.

## Abbreviations

MR-proADM: Mid-regional pro-adrenomedullin
ADM: Adrenomedullin
PLR: Positive likelihood ratio
NLR: Negative likelihood ratio
DOR: Diagnostic ratio
CI: Confidence interval
SROC: Summary receiver operator characteristic curves
CRP: C reactive protein
PCT: Procalcitonin

## References

[1] Angus DC, van der Poll T (2013). Severe sepsis and septic shock. N Engl J Med.

[2] Angus DC, Linde-Zwirble WT, Lidicker J, Clermont G, Carcillo J, Pinsky MR (2001). Epidemiology of severe sepsis in the United States: analysis of incidence, outcome, and associated costs of care. Crit Care Med.

[3] Martin GS, Mannino DM, Eaton S, Moss M (2003). The epidemiology of sepsis in the United States from 1979 through 2000. N Engl J Med.

[4] Wacker C, Prkno A, Brunkhorst FM, Schlattmann P (2013). Procalcitonin as a diagnostic marker for sepsis: a systematic review and meta-analysis. Lancet Infect Dis.

[5] Rangel-Frausto MS, Pittet D, Costigan M, Hwang T, Davis CS, Wenzel RP (1995). The natural history of the Systemic Inflammatory Response Syndrome (SIRS). Aprospect Study Jama.

[6] Standage SW, Wong HR (2011). Biomarkers for pediatric sepsis and septic shock. Expert Rev Anti Infect Ther.

[7] van Rossum AM, Wulkan RW, Oudesluys-Murphy AM (2004). Procalcitonin as an early marker of infection in neonates and children. Lancet Infect Dis.

[8] Chenevier-Gobeaux C, Borderie D, Weiss N, Mallet-Coste T, Claessens YE (2015). Presepsin (sCD14-ST), an innate immune response marker in sepsis. Clin Chim Acta.

[9] Piccioni A, Santoro MC, de Cunzo T, Tullo G, Cicchinelli S, Saviano A (2021). Presepsin as early marker of sepsis in emergency department: a narrative review. Medicina (Kaunas).

[10] Xiao HL, Wang GX, Wang Y, Tan ZM, Zhou J, Yu H (2022). Dynamic blood presepsin levels are associated with severity and outcome of acute pancreatitis: A prospective cohort study. World J Gastroenterol.

[11] Hayashi M, Yaguchi Y, Okamura K, Goto E, Onodera Y, Sugiura A (2017). A case of extensive burn without sepsis showing high level of plasma presepsin (sCD14-ST). Burns Open.

[12] Saeed K, Legramante JM, Angeletti S, Curcio F, Miguens I, Poole S (2021). Mid-regional pro-adrenomedullin as a supplementary tool to clinical parameters in cases of suspicion of infection in the emergency department. Expert Rev Mol Diagn.

[13] Gille J, Ostermann H, Dragu A, Sablotzki A (2017). MR-proADM: a new biomarker for early diagnosis of sepsis in burned patients. J Burn Care Res.

[14] McInnes MDF, Moher D, Thombs BD, McGrath TA, Bossuyt PM, and the P-DTAG (2018). Preferred reporting items for a systematic review and meta-analysis of diagnostic test accuracy studies: the PRISMA-DTA statement. JAMA.

[15] Whiting PF, Rutjes AW, Westwood ME, Mallett S, Deeks JJ, Reitsma JB (2011). QUADAS-2: a revised tool for the quality assessment of diagnostic accuracy studies. Ann Intern Med.

[16] Lee S, Song J, Park DW, Seok H, Ahn S, Kim J (2022). Diagnostic and prognostic value of presepsin and procalcitonin in non-infectious organ failure, sepsis, and septic shock: a prospective observational study according to the Sepsis-3 definitions. BMC Infect Dis.

[17] Kang ES, Lee JH (2022). Diagnostic value of presepsin in odontogenic infection: a retrospective study. Maxillofac Plast Reconstr Surg.

[18] Jeong YK, Kim EY (2022). Predictive role of changes in presepsin and early sepsis in ICU Patients after abdominal surgery. J Surg Res.

[19] Tahmaz A, ÖZtoprak N, Kizilates F. Investigation of Plasma Preseptin (sCD14-ST) Levels in Sepsis. Mediterranean Journal of Infection Microbes and Antimicrobials. 2022;11:11.

[20] Chen J, Huang ZB, Li H, Zheng X, Chen JJ, Wang XB (2021). Early diagnostic biomarkers of sepsis for patients with acute-on-chronic liver failure: a multicenter study. Infect Dis Ther.

[21] Yonaha T, Maruta T, Otao G, Igarashi K, Nagata S, Yano T (2021). The diagnostic and prognostic value of mature and total adrenomedullin for sepsis: a prospective observational study. Anaesthesiol Intensive Ther.

[22] Abdelshafey EE, Nasa P, Elgohary AE, Khalil MF, Rashwan MA, Ghezala HB (2021). Role of presepsin for the diagnosis of sepsis and ICU mortality: a prospective controlled study. Indian J Crit Care Med.

[23] Liu C, Qiu Q (2020). Diagnostic value of PCT, SAA and presepsin in acute sepsis patients. Int J Clin Exp Med.

[24] Yamamoto T, Nishimura T, Kaga S, Uchida K, Tachibana Y, Esaki M (2019). Diagnostic accuracy of presepsin for sepsis by the new Sepsis-3 definitions. Am J Emerg Med.

[25] Nakamura Y, Hoshino K, Kiyomi F, Kawano Y, Mizunuma M, Tanaka J (2019). Comparison of accuracy of presepsin and procalcitonin concentrations in diagnosing sepsis in patients with and without acute kidney injury. Clin Chim Acta.

[26] Juroš GF, Nikić MT, Šarić SD, Perić M, Rogić D (2019). Contribution of presepsin, procalcitonin and C-reactive protein to the SOFA score in early sepsis diagnosis in emergency abdominal surgical patients. Signa Vitae: J Intesive Care Emerg Med.

[27] Jereb M, Mavric M, Skvarc M, Drobnic A, Dolenc S, Strunjas NP (2019). Usefulness of presepsin as diagnostic and prognostic marker of sepsis in daily clinical practice. J Infect Dev Ctries.

[28] Lu B, Zhang Y, Li C, Liu C, Yao Y, Su M (2018). The utility of presepsin in diagnosis and risk stratification for the emergency patients with sepsis. Am J Emerg Med.

[29] Li H, Wu S (2018). Prognostic value of soluble cluster differentiation antigen 14 subtype and platelet activating factor in patients with septic shock. Int J Clin Exp Med.

[30] El-Shafie ME-S, Taema KM, El-Hallag MM, Kandeel AMA (2017). Role of presepsin compared to C-reactive protein in sepsis diagnosis and prognostication. Egypt J Critical Care Med.

[31] de Guadiana Romualdo LG, Torrella PE, Acebes SR, Otón MDA, Sánchez RJ, Holgado AH (2017). Diagnostic accuracy of presepsin (sCD14-ST) as a biomarker of infection and sepsis in the emergency department. Clin Chim Acta.

[32] Klouche K, Cristol JP, Devin J, Gilles V, Kuster N, Larcher R (2016). Diagnostic and prognostic value of soluble CD14 subtype (Presepsin) for sepsis and community-acquired pneumonia in ICU patients. Ann Intensive Care.

[33] Ali FT, Ali MA, Elnakeeb MM, Bendary HN (2016). Presepsin is an early monitoring biomarker for predicting clinical outcome in patients with sepsis. Clin Chim Acta.

[34] Amer HA, Ghareeb H, Lotfy NM, El-Azizi NO, Mahmoud AM (2016). Presepsin a diagnostic marker for sepsis in intensive care unit patients. Egypt J Immunol.

[35] Enguix-Armada A, Escobar-Conesa R, García-De La Torre A, De La Torre-Prados MV (2016). Usefulness of several biomarkers in the management of septic patients: C-reactive protein, procalcitonin, presepsin and mid-regional pro-adrenomedullin. Clin Chem Lab Med (CCLM).

[36] Sato M, Takahashi G, Shibata S, Onodera M, Suzuki Y, Inoue Y (2015). Clinical performance of a new soluble CD14-subtype immunochromatographic test for whole blood compared with chemiluminescent enzyme immunoassay: use of quantitative soluble CD14-subtype immunochromatographic tests for the diagnosis of sepsis. PLoS ONE.

[37] Carpio R, Zapata J, Spanuth E, Hess G (2015). Utility of presepsin (sCD14-ST) as a diagnostic and prognostic marker of sepsis in the emergency department. Clin Chim Acta.

[38] Sargentini V, Ceccarelli G, D’Alessandro M, Collepardo D, Morelli A, D’Egidio A (2015). Presepsin as a potential marker for bacterial infection relapse in critical care patients. a preliminary study. Clin Chem Lab Med (CCLM).

[39] Popa TO, Cimpoesu D, Dorobat CM (2015). Diagnostic and prognostic value of presepsin in the emergency department. Rev Med Chir Soc Med Nat Iasi.

[40] Behnes M, Bertsch T, Lepiorz D, Lang S, Trinkmann F, Brueckmann M (2014). Diagnostic and prognostic utility of soluble CD 14 subtype (presepsin) for severe sepsis and septic shock during the first week of intensive care treatment. Crit Care.

[41] Madenci ÖÇ, Yakupoğlu S, Benzonana N, Yücel N, Akbaba D, Kaptanağası AO (2014). Evaluation of soluble CD14 subtype (presepsin) in burn sepsis. Burns.

[42] Kweon OJ, Choi J-H, Park SK, Park AJ (2014). Usefulness of presepsin (sCD14 subtype) measurements as a new marker for the diagnosis and prediction of disease severity of sepsis in the Korean population. J Crit Care.

[43] Liu B, Chen YX, Yin Q, Zhao YZ, Li CS (2013). Diagnostic value and prognostic evaluation of Presepsin for sepsis in an emergency department. Crit Care.

[44] Vodnik T, Kaljevic G, Tadic T, Majkic-Singh N (2013). Presepsin (sCD14-ST) in preoperative diagnosis of abdominal sepsis. Clin Chem Lab Med.

[45] Ulla M, Pizzolato E, Lucchiari M, Loiacono M, Soardo F, Forno D (2013). Diagnostic and prognostic value of presepsin in the management of sepsis in the emergency department: a multicenter prospective study. Crit Care.

[46] Shozushima T, Takahashi G, Matsumoto N, Kojika M, Okamura Y, Endo S (2011). Usefulness of presepsin (sCD14-ST) measurements as a marker for the diagnosis and severity of sepsis that satisfied diagnostic criteria of systemic inflammatory response syndrome. J Infect Chemother.

[47] Venugopalan DP, Pillai G, Krishnan S (2019). Diagnostic value and prognostic use of presepsin versus procalcitonin in sepsis. Cureus.

[48] Zhenyu L, Huajie Z, Jun Z, BIn C (2016). Early diagnostic value and prognostic significance of serum presepsin (sCD14-ST) in sepsis. Chin J Emerg Med.

[49] Martin-Fernandez M, Vaquero-Roncero LM, Almansa R, Gómez-Sánchez E, Martín S, Tamayo E (2020). Endothelial dysfunction is an early indicator of sepsis and neutrophil degranulation of septic shock in surgical patients. BJS open.

[50] Angeletti S, Spoto S, Fogolari M, Cortigiani M, Fioravanti M, De Florio L (2015). Diagnostic and prognostic role of procalcitonin (PCT) and MR-pro Adrenomedullin (MR-pro ADM) in bacterial infectious. APMIS.

[51] Angeletti S, Battistoni F, Fioravanti M, Bernardini S, Dicuonzo G (2013). Procalcitonin and mid-regional pro-adrenomedullin test combination in sepsis diagnosis. Clin Chem Lab Med.

[52] Angeletti S, Dicuonzo G, Fioravanti M, De Cesaris M, Fogolari M, Lo Presti A (2015). Procalcitonin, MR-proadrenomedullin, and cytokines measurement in sepsis diagnosis: advantages from test combination. Dis Markers.

[53] Spoto S, Nobile E, Carna EPR, Fogolari M, Caputo D, De Florio L (2020). Best diagnostic accuracy of sepsis combining SIRS criteria or qSOFA score with Procalcitonin and Mid-Regional pro-Adrenomedullin outside ICU. Sci Rep.

[54] Spoto S, Cella E, de Cesaris M, Locorriere L, Mazzaroppi S, Nobile E (2018). Procalcitonin and MR-proadrenomedullin combination with SOFA and qSOFA scores for sepsis diagnosis and prognosis: a diagnostic algorithm. Shock.

[55] Simon L, Saint-Louis P, Amre DK, Lacroix J, Gauvin F (2008). Procalcitonin and C-reactive protein as markers of bacterial infection in critically ill children at onset of systemic inflammatory response syndrome. Pediatr Crit Care Med.

[56] Luzzani A, Polati E, Dorizzi R, Rungatscher A, Pavan R, Merlini A (2003). Comparison of procalcitonin and C-reactive protein as markers of sepsis. Crit Care Med.

[57] Tan M, Lu Y, Jiang H, Zhang L (2019). The diagnostic accuracy of procalcitonin and C-reactive protein for sepsis: A systematic review and meta-analysis. J Cell Biochem.

[58] Okamura Y, Yokoi H (2011). Development of a point-of-care assay system for measurement of presepsin (sCD14-ST). Clin Chim Acta.

[59] Valenzuela-Sánchez F, Valenzuela-Méndez B, Rodríguez-Gutiérrez JF, Estella-García Á, González-García M (2016). New role of biomarkers: mid-regional pro-adrenomedullin, the biomarker of organ failure. Ann Transl Med.

[60] Dou YH, Du JK, Liu HL, Shong XD (2013). The role of procalcitonin in the identification of invasive fungal infection-a systemic review and meta-analysis. Diagn Microbiol Infect Dis.

[61] Jeong S, Park Y, Cho Y, Kim HS (2012). Diagnostic utilities of procalcitonin and C-reactive protein for the prediction of bacteremia determined by blood culture. Clin Chim Acta.

[62] Bamba Y, Moro H, Aoki N, Koizumi T, Ohshima Y, Watanabe S (2018). Increased presepsin levels are associated with the severity of fungal bloodstream infections. PLoS ONE.

[63] Nockher WA, Wick M, Pfister HW (1999). Cerebrospinal fluid levels of soluble CD14 in inflammatory and non-inflammatory diseases of the CNS: upregulation during bacterial infections and viral meningitis. J Neuroimmunol.

[64] Decker SO, Krüger A, Wilk H, Grumaz S, Vainshtein Y, Schmitt FCF (2019). New approaches for the detection of invasive fungal diseases in patients following liver transplantation-results of an observational clinical pilot study. Langenbecks Arch Surg.

[65] Sozio E, Tascini C, Fabris M, D'Aurizio F, De Carlo C, Graziano E (2021). MR-proADM as prognostic factor of outcome in COVID-19 patients. Sci Rep.

[66] Michels M, Djamiatun K, Faradz SM, Koenders MM, de Mast Q, van der Ven AJ (2011). High plasma mid-regional pro-adrenomedullin levels in children with severe dengue virus infections. J Clin Virol.

[67] Valenzuela Sanchez F, Valenzuela Mendez B, Rodríguez Gutierrez J, de Bohollo Austria R, Rubio Quiñones J, Puget Martínez L (2015). Initial levels of mr-proadrenomedullin: a predictor of severity in patients with influenza a virus pneumonia. Int Care Med Exp.

[68] Yang HS, Hur M, Yi A, Kim H, Lee S, Kim SN (2018). Prognostic value of presepsin in adult patients with sepsis: Systematic review and meta-analysis. PLoS ONE.

[69] Singer M, Deutschman CS, Seymour CW, Shankar-Hari M, Annane D, Bauer M (2016). The third international consensus definitions for sepsis and septic shock (Sepsis-3). JAMA.

[70] Wu J, Hu L, Zhang G, Wu F, He T (2015). Accuracy of presepsin in sepsis diagnosis: a systematic review and meta-analysis. PLoS ONE.

[71] Ozdemir AA, Elgormus Y (2017). Diagnostic value of presepsin in detection of early-onset neonatal sepsis. Am J Perinatol.

[72] Poggi C, Bianconi T, Gozzini E, Generoso M, Dani C (2015). Presepsin for the detection of late-onset sepsis in preterm newborns. Pediatrics.

[73] Montaldo P, Rosso R, Santantonio A, Chello G, Giliberti P (2017). Presepsin for the detection of early-onset sepsis in preterm newborns. Pediatr Res.

[74] Masson S, Caironi P, Fanizza C, Thomae R, Bernasconi R, Noto A (2015). Circulating presepsin (soluble CD14 subtype) as a marker of host response in patients with severe sepsis or septic shock: data from the multicenter, randomized ALBIOS trial. Intensive Care Med.

[75] Xiao H, Wang G, Wang Y, Tan Z, Sun X, Zhou J (2022). Potential value of presepsin guidance in shortening antibiotic therapy in septic patients: a multicenter prospective cohort Trial. Shock.

