# Supplementary figures and images for: Comparison of presepsin and Mid-regional pro-adrenomedullin in the diagnosis of sepsis or septic shock: a systematic review and meta-analysis
Source: BMC Infect Dis. 2023 May 5;23:288. doi: 10.1186/s12879-023-08262-4 (PMC10160726; doi:10.1186/s12879-023-08262-4)

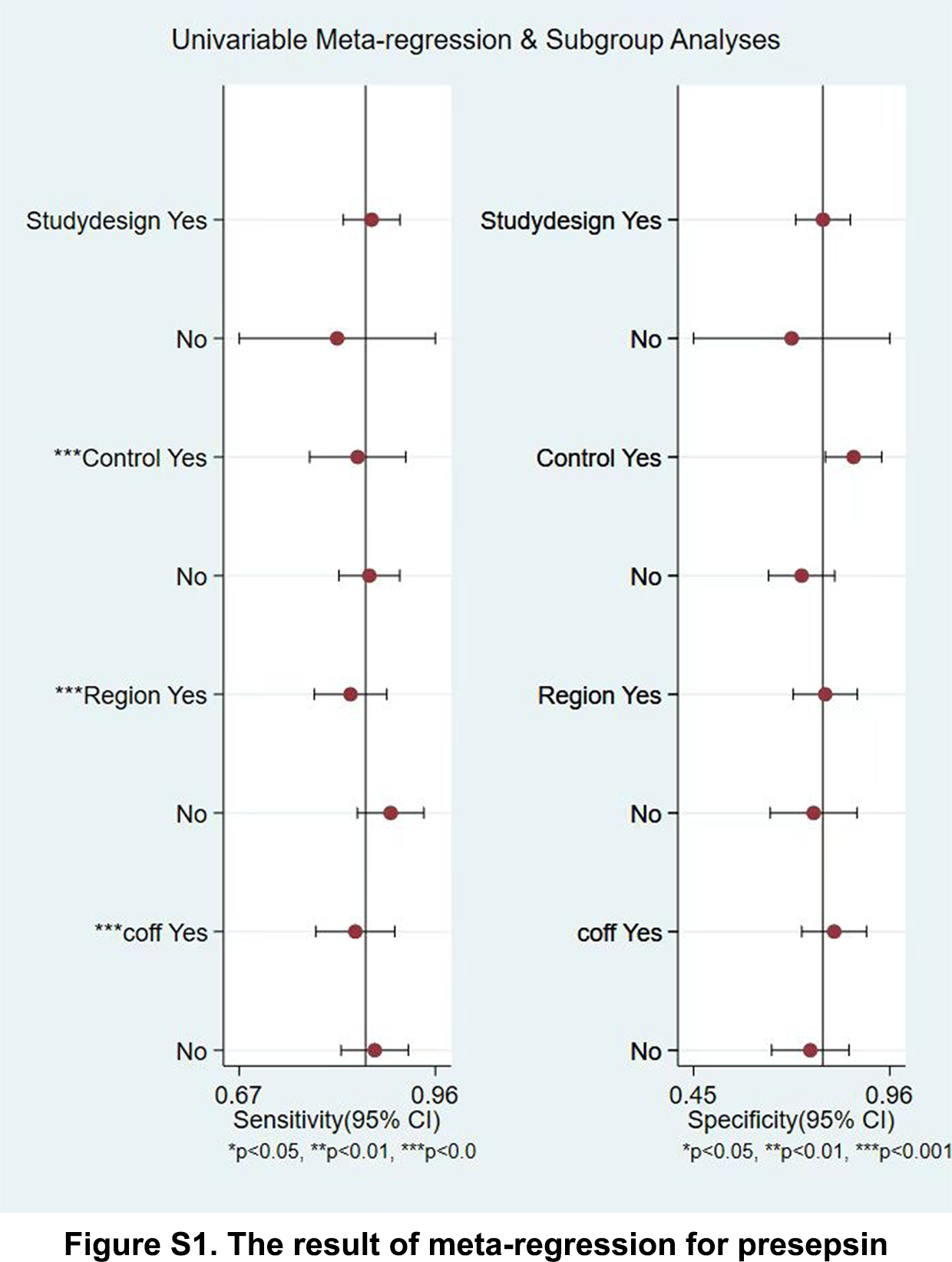

Supplement: Supplementary file 1 — Additional file 1: Figure S1. The result of meta-regression for presepsin. [file 12879_2023_8262_MOESM1_ESM.tif]

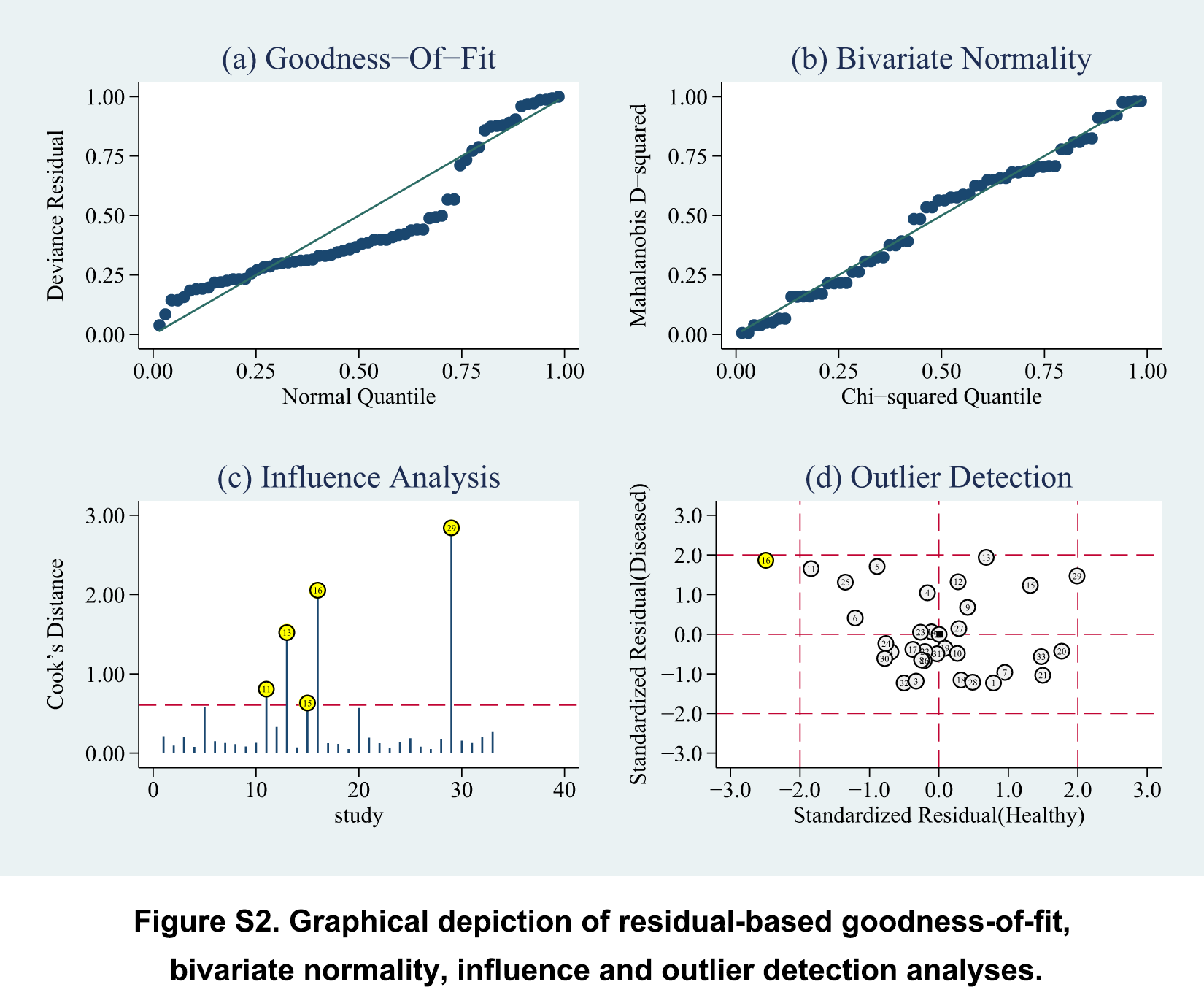

Supplement: Supplementary file 2 — Additional file 2: Figure S2. Graphical depiction of residual-based goodness-of-fit, bivariate normality, influence and outlier detection analyses. [file 12879_2023_8262_MOESM2_ESM.tif]

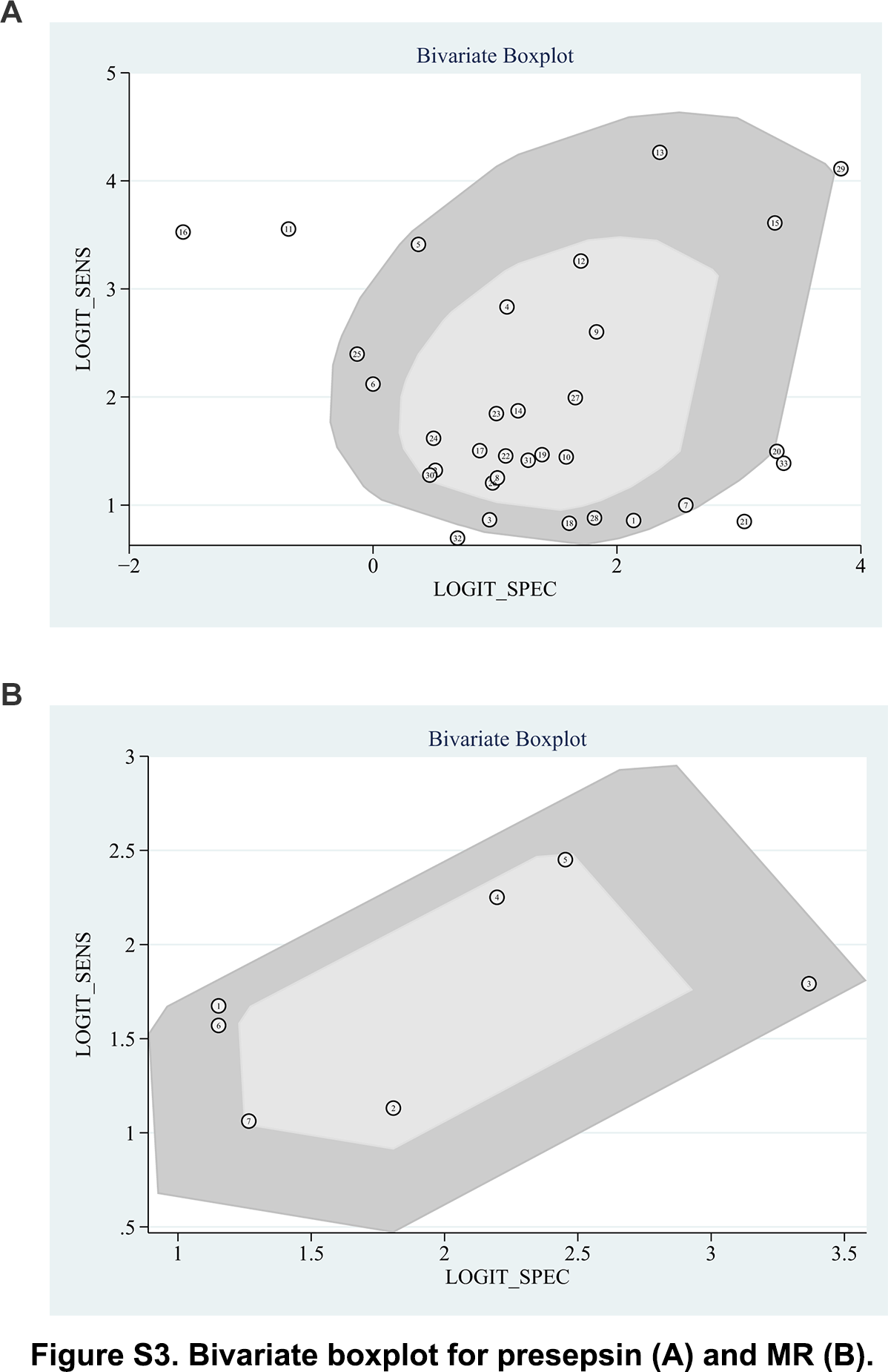

Supplement: Supplementary file 3 — Additional file 3: Figure S3. Bivariate boxplot for presepsin (A) and MR (B). [file 12879_2023_8262_MOESM3_ESM.tif]

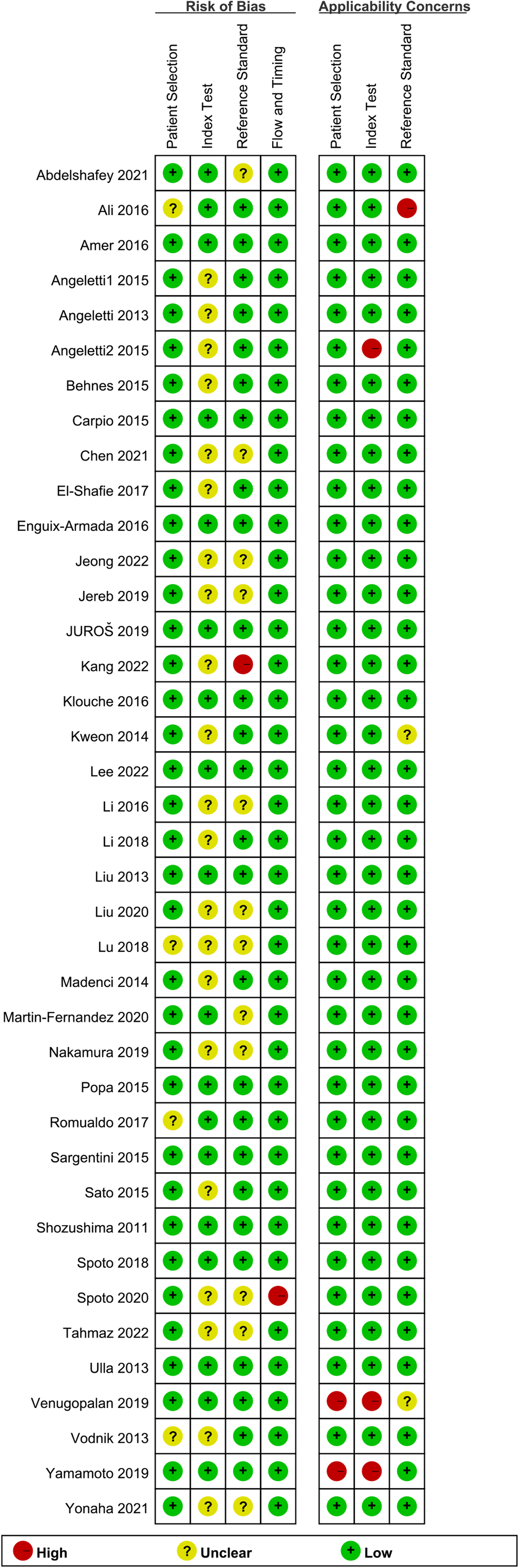

Supplement: Supplementary file 5 — Additional file 5: Quality assessment of the included studies. [file 12879_2023_8262_MOESM5_ESM.tif]
