# Supplementary material for: Comparison of presepsin and Mid-regional pro-adrenomedullin in the diagnosis of sepsis or septic shock: a systematic review and meta-analysis
Source: BMC Infect Dis. 2023 May 5;23:288. doi: 10.1186/s12879-023-08262-4 (PMC10160726; doi:10.1186/s12879-023-08262-4)
Supplement: Supplementary file 4 — Additional file 4: Table S1. Search strategy used in this study [file 12879_2023_8262_MOESM4_ESM.docx]

Table S1. Search strategy used in this study

| **database** | **Search stratety** | **Item found** |
| --- | --- | --- |
| PubMed | ("sepsis"[MeSH Terms] OR sepsis[Title/Abstract] OR "septic shock"[Title/Abstract]) AND ((presepsin[Title/Abstract]) OR ("sCD14-ST"[Title/Abstract]) OR ("soluble CD14 subtype"[Title/Abstract]) OR ("P-SEP"[Title/Abstract]) OR ("mid-regional pro-adrenomedullin"[Title/Abstract]) OR ("MR pro-ADM"[Title/Abstract]) OR ("MR-proADM"[Title/Abstract])) AND (adult) | 181 |
| Embase | (('sepsis':ab,ti) OR ('pyemia':ab,ti) OR ('septic shock':ab,ti)) AND (('presepsin':ab,ti) OR ('sCD14-ST':ab,ti) OR ('soluble CD14 subtype':ab,ti) OR ('P-SEP':ab,ti) OR ('mid-regional pro-adrenomedullin':ab,ti) OR ('MR pro-ADM':ab,ti) OR ('MR-proADM':ab,ti)) AND ('adult'/de) | 282 |
| Web of Science | ((TI="sepsis" OR AB="sepsis") OR (TI="pyemia" OR AB="pyemia") OR (TI="septic shock" OR AB="septic shock")) AND ((TI="presepsin" OR AB="presepsin") OR (TI="sCD14-ST" OR AB="sCD14-ST") OR (TI="soluble CD14 subtype" OR AB="soluble CD14 subtype") OR (TI="P-SEP" OR AB="P-SEP") OR (TI="mid-regional pro-adrenomedullin" OR AB="mid-regional pro-adrenomedullin") OR (TI="MR pro-ADM" OR AB="MR pro-ADM") OR (TI="MR-proADM" OR AB="MR-proADM")) AND (TS="adult") | 119 |
| CNKI | (TI='sepsis' OR AB='sepsis') AND ((TI='presepsin' OR AB='presepsin') OR (TI='sCD14-ST' OR AB='sCD14-ST') OR (TI='soluble CD14 subtype' OR AB='soluble CD14 subtype') OR (TI='P-SEP' OR AB='P-SEP')) AND ((FT='adult') OR (FT='adult')) | 32 |
| wanfang | (TI:'sepsis' OR AB:'sepsis') AND ((TI:'presepsin' OR AB:'presepsin') OR (TI:'sCD14-ST' OR AB:'sCD14-ST') OR (TI:'soluble CD14 subtype' OR AB:'soluble CD14 subtype') OR (TI:'P-SEP' OR AB:'P-SEP')) | 19 |
